# The Induction of APC with a Distinct Tolerogenic Phenotype via Contact-Dependent STAT3 Activation

**DOI:** 10.1371/journal.pone.0006846

**Published:** 2009-08-31

**Authors:** Devorah Gur-Wahnon, Zipora Borovsky, Meir Liebergall, Jacob Rachmilewitz

**Affiliations:** 1 Goldyne Savad Institute of Gene Therapy, Hadassah-Hebrew University Medical Center, Jerusalem, Israel; 2 Department of Orthopedic Surgery, Hadassah-Hebrew University Medical Center, Jerusalem, Israel; New York University School of Medicine, United States of America

## Abstract

**Background:**

Activation of the signal transducer and activator of transcription 3 (STAT3) within antigen presenting cells (APCs) is linked to abnormal APCs differentiation and function. We have previously shown that STAT3 is activated within APC by a novel contact-dependent mechanism, which plays a key role in mediating the immunomodulatory effects of hMSC. In order to better understand the underlying mechanisms that control APC maturation in a contact dependent manner, we extended our observation to tumor cells. Tumors were shown to secrete a variety of tumor-derived factors that activate STAT3 within infiltrating APCs. We now tested whether tumor cells can activate STAT3 within APC using the contact-dependent mechanism, in addition to soluble factors, and compared these two STAT3 activating pathways.

**Principal Findings:**

We demonstrate that in addition to tumor-derived secreted factors tumor cells activate STAT3 by a mechanism that is based on cell-cell interaction. We further demonstrate that these two STAT3 activating mechanisms differ in their JAK usage and their susceptibility to JSI-124 inhibition thereby representing two distinct pathways. Significantly, although both pathways activate STAT3, they modulate DCs maturation in a different manner that results in disparate phenotypic outcomes. Whereas the soluble-dependent pathway results in an immature phenotype, the contact-dependent pathway results in an apparently mature phenotype. Albeit their mature-like phenotype these latter cells express the tolerogenic markers ILT3 and ILT4 and possess T cell inhibitory activity.

**Significance:**

This data suggests that, in at least certain cellular microenvironments, cell:cell interactions represent a novel way to activate STAT3 signaling, uncouple APC activation events and consequently regulate immunity and tolerance. Significantly, we have now demonstrated that this contact-dependent signaling pathway differs from that mediated by soluble factors and cytokines, inducing disparate phenotypic outcome, suggesting these two mechanisms have different and possibly complementary biological functions.

## Introduction

Antigen-presenting cells (APCs), and specifically dendritic cells (DCs), are the most potent inducers of the immune response. DCs in the periphery capture and process antigens in their immature state followed by a maturation process in response to a spectrum of stimuli, allowing them to induce both innate and adaptive responses [1]. Only upon receiving maturation signals, DCs migrate to lymphoid organs, secrete cytokines and express co-stimulatory molecules that are required for lymphocyte activation [1]. In recent years, however, there is growing evidence suggesting that DCs not only initiate T cell responses but are also involved in silencing T cell immune responses. These functions of DCs are thought to be mainly dependent on their activation and differentiation state. For example, terminally differentiated mature DCs can efficiently induce the development of effector T cells, whereas immature DCs or partially matured DCs are involved in maintenance of peripheral tolerance. Hence, APCs and specifically DCs orchestrate a range of immune responses including induction and suppression of T cell activation [1], [2].

Regulation of DCs maturation occurs through the function of Janus activated kinase (JAK)/signal transducer and activator of transcription (STAT) signaling pathway [3]. The JAK family of tyrosine kinases and STAT are important components of diverse signal transduction pathways that are actively involved in cellular survival, proliferation, differentiation and apoptosis. Four members of the Jak family have been identified in mammalian cells, Jak1, Jak2, Jak3 and Tyk2 [4]. Cytokine receptor-ligand binding induces receptor oligomerization and phophorylation, followed by Jak activation. Activated Jaks phosphorylate receptors on target tyrosine residues, generating docking sites for STATs, which are subsequently recruited and phosphorylated by activated Jaks. Dimerized STATs then translocate to the nucleus, where they modulate expression of target genes [5]. One of these proteins STAT3, has been implicated as a negative regulator of the immune response [6]. Mice devoid of the STAT3 gene in macrophages and neutrophils have enhanced inflammatory activity, leading to the development of chronic colitis [6].

STAT3 has been recently proposed as an important molecule that mediates tumor induced immunosupression. STAT3 is constitutively active in many tumor cells and was found to have an important role in oncogenesis [7]. In addition STAT3 was found to have a profound role in regulating the immune responses in the tumor micro-environment. In tumor cells, STAT3 activation has been linked to both inhibition of pro-inflammatory cytokine secretion and induction of anti-inflammatory cytokine secretion, such as IL-10 and VEGF [8]. These latter anti-inflammatory cytokines can, in turn, induce STAT3 activation within neighboring DCs, thereby influencing their functional maturation [8]. Collectively, tumor cells were shown to secrete soluble factors that activate STAT3 and suppress DCs function.

An effective anti-tumor immune response requires the participation of the host APCs, mostly DCs and macrophages [7]. Macrophages were shown to be recruited to the tumor site by cytokines, chemokines and other tumor derived factors, and once within the tumor environment they are skewed toward an anti-inflammatory phenotype and thereby promote tumor growth [9]. DCs in the tumor microenvironment are usually found in an immature state, characterized by an insufficient level of expression of MHC class II complexes, co-stimulatory molecules such as CD80 and CD86 and IL-12. These cells are not only poor antigen-presenting cells, but could further induce immune tolerance [10], [11]. Tolerogenic DCs are generated in the absence of inflammatory mediators, and recent studies have shown that DCs can be further blocked from becoming effective antigen presenting cells by tumor secreted factors that inhibit their maturation [10], [11].

We have previously described that human Mesenchymal Stem Cells (hMSC) inhibit T cells indirectly by contact-dependent induction of regulatory APCs (both monocytes and DCs) with T cell suppressive properties [12]. Accordingly, when APCs came into productive contact with hMSC, the secretion of pro-inflammatory cytokines such as TNF-α and IL-12 were inhibited while the anti-inflammatory cytokine IL-10 was induced. Under these circumstances, IFN-γ secretion and T- cell proliferation were inhibited [12]. This contact-dependent induction of regulatory APCs was dependent on the activation of STAT3 in these cells and could be blocked with specific STAT3 inhibitors [13]. In the present study, we demonstrated that tumor cells modulate APCs function by using two parallel mechanisms for the activation of STAT3; the first involving secretion of soluble cytokines as previously described [8], [14], and second involving a novel pathway that is based on cell-cell interaction. While these two distinct pathways activate STAT3 within APCs, they result in disparate phenotypic outcomes. A contact-dependent mechanism may provide a molecular explanation for how the surrounding microenvironment influences APCs maturation in a focused and unique way.

## Results

### Cancer cells inhibit TNF-α and IFN-γ secretion while inducing IL-10 production

Recent studies have suggested that tumor cells secrete tumor-derived factors that induce STAT3 activity within neighboring APCs, effecting their maturation and function [8]. Based upon our previous insight into a contact-dependent effect of hMSC on APC phenotype and function [12], along with the linking of this effect to STAT3 activation [13], we now tested whether a similar contact-dependent STAT3 activation is also induced in tumor cell-APC interaction.

We started by co-culturing two different tumor cell lines (MCF-7 breast carcinoma; PC3 prostate carcinoma) with a mixture of monocytes, T cells, and the superantigen SEB as activator. Both tumor cell lines successfully inhibited IFN-γ and TNF-α secretion (Supporting data Fig. S1A) and promoted IL-10 secretion, with the latter, primarily produced by the monocytes, as determined using immunofluorescence and flow cytometry (Supporting data Fig. S1B).

### Increased STAT3 activity in monocytes:tumor cells co-cultures is dependent on both cell:cell contact and tumor cells secreted factor(s)

**Figure 1:**
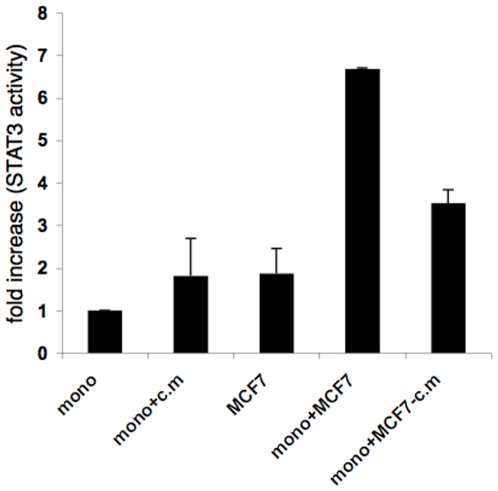
Increased STAT3 activity in co-cultures is dependent on both cell:cell contact and secreted factor(s). Confluent cultures of MCF-7 (2.5×10^4^/well) in a 24 well plate were incubated for 24 hours, then the cells were either left untreated or their conditioned media was collected and replaced with fresh media (without conditioned medium –c.m). Monocytes (2.5×10^4^ cells/well) were either added to tumor cell's cultures (both untreated and cells that received fresh media-c.m.) or were incubated alone or with tumor cell's conditioned medium (c.m). After 2 hours, nuclear extracts were prepared and tested for STAT3 activity using Non-Radioactive STAT3 Transcription Factor Assay. Comparable results were obtained in two independent experiments.

**Figure 2:**
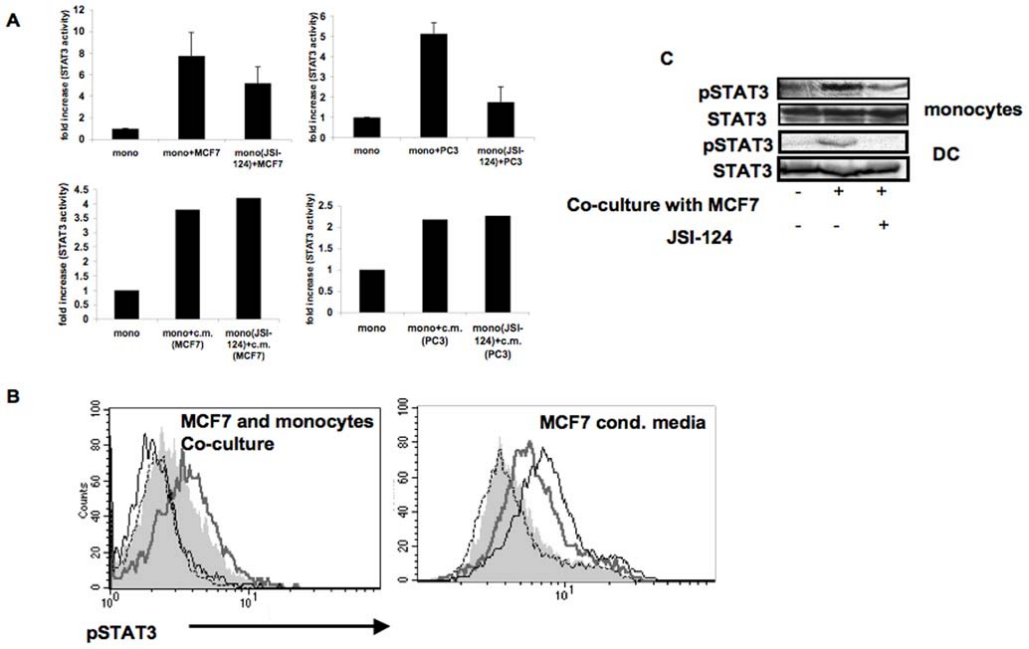
Contact-dependent but not conditioned media-induced STAT3 activity is sensitive to JSI-124 inhibition. A. Monocytes were either left untreated or treated with JSI-124 (10 µM) for 1 hour at 37°C, and then washed. Treated and untreated cells were then cultured alone or were co-cultured with either MCF-7 or PC3 cells (upper panels) or with their conditioned media (lower panels). After 2 hours, nuclear extracts were prepared and tested for STAT3 activity using Non-Radioactive STAT3 Transcription Factor Assay. Comparable results were obtained in four separate experiments. B. Monocytes were either left untreated or treated with JSI-124 (10 µM) for 1 hour at 37°C, and then washed. JSI-124-treated and untreated cells were then cultured alone or co-cultured together with tumor cells (MCF-7) or with tumor cell's conditioned media. After 2 hours, cells were harvested and phosphorylated-STAT3 was detected by intracellular immunostaining and flow cytometric analysis. The two cell types were distinguished based on their forward and side scatter characteristics and phosphorylated STAT3 staining in monocyte population is shown. Dashed line: isotype control; Filled grey area: monocytes alone; Thick grey line: monocytes that were either co-cultured with MCF-7 (left panel) or were cultured in MCF-7 conditioned media (right panel); Black line: JSI-124-treated monocytes incubated with MCF-7 or their conditioned media, as above. Comparable results were obtained in three independent experiments. C. Monocytes or DCs were either left untreated or treated with JSI-124 (as indicated above), and then were co-cultured with MCF-7, for 2 hours. Untreated cells were obtained by mixing MCF-7 and either monocytes or DC without incubation. Cell extracts were subjected to SDS-PAGE and immunoblotting of anti-phosphorylated STAT3 demonstrate induction of STAT3 and that JSI-124 treatment blocks contact-dependent STAT3 phosphorylation. Anti-STAT3 immunoblotting reveals relative amounts of protein in each lane (lower panels).

We next assessed STAT3 activity in these co-cultures using STAT3 transcription factor assay. As shown in figure 1, we observed an increase in STAT3, but not STAT1 (not shown), activity in tumor cells-monocyte co-cultures, as compared to each cell type cultured individually. We further assessed the contribution of soluble and contact-dependent factors to the increase in STAT3 activation in tumor cell-APC co-cultures. There is soluble factor-dependence of STAT3 induced activity in the tumor cells-monocyte co-cultures based on the increase in STAT3 activity in monocytes when both PC3 and MCF-7 were replaced with their conditioned media (Fig. 1 and 2A, lower panels), or when monocytes and the tumor cells were on opposite sides of a transwell membrane (not shown). However, the effect of conditioned media was lower than the combined effect achieved when the two cell types were co-cultured (Fig. 1 and 2A). Therefore, to eliminate interference from tumor cytokines in the co-cultures, tumor cell conditioned media was washed away just prior to adding the monocytes. Under these conditions, we observed significant increase in STAT3 activity after co-culturing the cells compared to its activity in each cell type cultured individually, measured 2 hours later. Suggesting that cell-cell contact may also contribute to STAT3 induction in addition to soluble factors (Fig. 1). Furthermore, intracellular staining for phosphorylated STAT3 revealed that this contact-dependent increase resulted from STAT3 activity within the monocytes (Fig. 2B).

### Contact-dependent but not conditioned media or IL-10-induced STAT3 activity and IL-10 production is sensitive to JSI-124 inhibition

**Figure 3:**
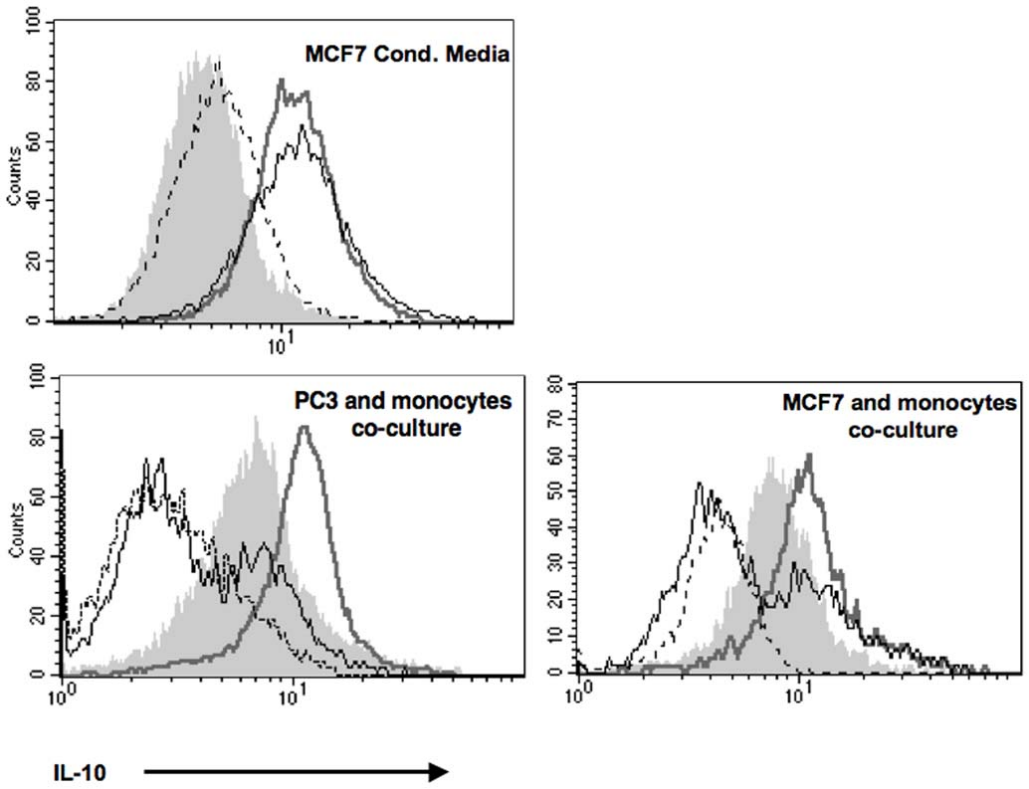
JSI-124 inhibits IL-10 production induced by cell:cell contact but not by tumor cell's conditioned media. Monocytes were either left untreated or treated with JSI-124 (10 µM) for 1 hour at 37°C, and then washed. JSI-124- treated and untreated cells were then cultured alone or co-cultured together with tumor cells (PC3 or MCF-7, as indicated; lower panels) or with tumor cell's conditioned media (upper panel). After 24 hours, cells were harvested and IL-10 production was detected by intracellular immunostaining and flow cytometric analysis. Monocytes that were gated based on their forward- and side-scatter characteristics are shown. Dashed line: Isotype control; Filled grey area: monocytes alone; Thick grey line: monocytes that were either co-cultured with tumor cells (lower panels) or were cultured in MCF-7 conditioned media (upper panel); Black line: JSI-124-treated monocytes incubated with tumor cells or their conditioned media, as above. Similar results were obtained in three independent experiments.

**Figure 4:**
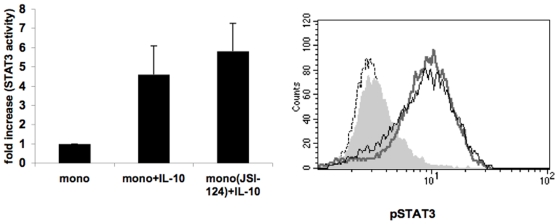
IL-10 induced STAT3 activity is insensitive to JSI-124 inhibition. JSI-124 treated and untreated monocytes (as described in figure 3) were cultured with or without IL-10 (10 ng/ml). After 2 hours, cells were harvested and either lysed and their nuclear extracts were tested for STAT3 activity using Non-Radioactive STAT3 Transcription Factor Assay (left panel) or phosphorylated STAT3 was detected by intracellular immunostaining and flow cytometric analysis (right panel). Dashed line: Isotype control; Filled grey line: monocytes alone; Thick grey line: IL-10 stimulated monocytes; Black line: IL-10 stimulated JSI-124 pretreated monocytes. Comparable results were obtained in three independent experiments.

Cytokine receptors that lack an intrinsic tyrosine kinase activity such as receptors for IL-10 and IL-6 rely on JAKs for initiating their signaling cascade and STAT3 activation (reviewed in [4]). Our previous data on hMSC:APC co-cultures [13] demonstrated that STAT3 activity is abrogated upon treatment with JSI-124 (cucurbitacin), a small natural molecule that inhibits JAK2 signaling [15], suggesting that the contact dependent mechanism may involve JAK2 signaling pathways. To further test this, we have pretreated monocytes with JSI-124, for 1 hour prior to their co-culturing with the tumor cells, or incubation with either IL-10 or tumor cell's conditioned media. Pretreatment of the cells was necessary in order to avoid JAK2/STAT3 inhibition in the tumor cells while focusing on signaling events within the APC. Pretreatment of monocytes with JSI-124, resulted in a significant reduction in STAT3 activity (measured by both STAT3 transcription factor assay (Fig. 2A, upper panels) or phospho-STAT3 intracellular immunostaining (Fig. 2B, left panel)) and in IL-10 production by the monocytes (Fig. 3, lower panels) in tumor cell:monocyte co-cultures. On the other hand, JSI-124 pre-treatment had no effect on STAT3 activity (Fig. 2A, lower panels), phospho-STAT3 immunostaining (Fig. 2B, right panel) or IL-10 production (Fig. 3, upper panel) when monocytes were incubated with MCF-7's conditioned media. Likewise, JSI-124 had no effect on STAT3 activity (Fig. 4A) or phospho-STAT3 intracellular immunostaining (Fig. 4B) in IL-10-treated monocytes. This activation of STAT3 as well as its sensitivity to JSI-124 treatment was further demonstrated using anti-phosphorylated STAT3 immunoblotting using both monocytes and DC co-cultured with MCF-7 cells (Fig. 2C). Hence, using these specific conditions (i.e. 1 h pre-treatment prior to stimulation) we could discriminate between the soluble factor- and contact- dependent STAT3 activating pathways. These results further support that in tumor-APC interaction there is a contact-dependent activation of STAT3 in APC, that unlike the parallel cytokine-mediated pathway, is sensitive to JSI-124 inhibition (as is the case with hMSC-APC co-cultures [13]).

### Cell:cell contact but not conditioned media or IL-10 induce JAK2 phosphorylation

**Figure 5:**
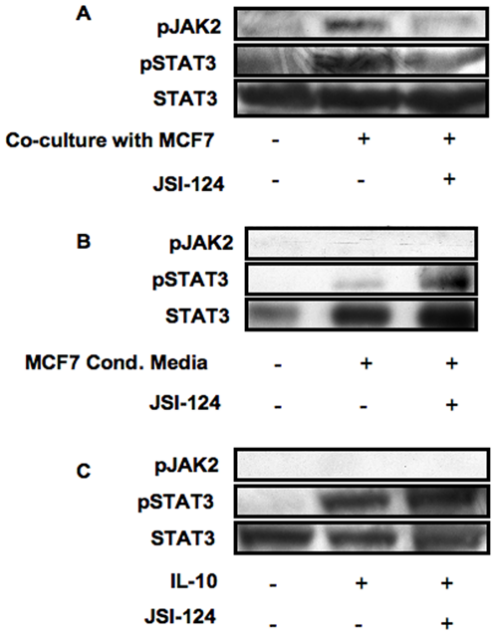
Cell:cell contact but not conditioned media or IL-10 induce JAK2 phosphorylation. Monocytes were either left untreated or treated with JSI-124 (as indicated above), and then were treated with either IL-10 (10 ng/ml), MCF-7's conditioned media or were co-cultured with MCF-7, for 2 hours. Untreated cells in the case of co-cultures were obtained by mixing MCF-7 and monocytes without incubation. Cell extracts were subjected to SDS-PAGE and anti-phosphorylated JAK2 immunoblotting (upper panels). Immunoblotting of anti-phosphorylated STAT3 demonstrates induction of STAT3 activation by all treatments regardless of JAK2 phosphorylation (middle panels) and that JSI-124 treatment selectively blocks contact- and JAK2-dependent STAT3 phosphorylation. Anti-STAT3 immunoblotting reveals relative amounts of protein in each lane (lower panels). Similar results were obtained in three independent experiments.

**Figure 6:**
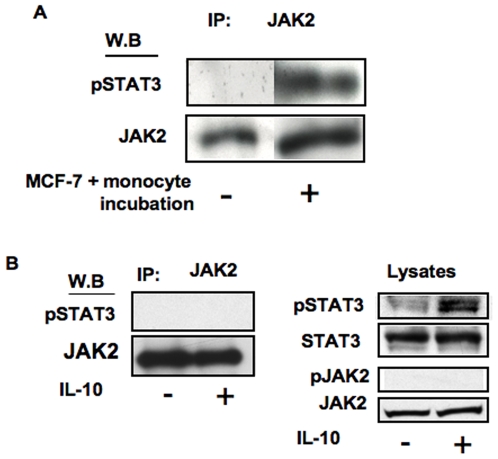
phosphorylated-STAT3 co-immunoprecipitates with JAK2 upon MCF7:monocyte cell contact but not IL-10 treatment. A: Monocytes were either co-cultured with MCF-7, for 2 hours or mixed with MCF-7 without incubation as a control, and then lysed. B: monocytes were either left untreated or were treated with IL-10 (10 ng/ml) for 2 hours, and then lysed. Cell extracts were subjected to immunoprecipitation by anti-JAK2 antibodies and the precipitates were separated on SDS-PAGE. Anti-phosphorylated STAT3 immunoblotting demonstrates that phosphorylated-STAT3 associates with JAK2 upon contact-dependent activation but not IL-10 treatment (upper panels). Anti-JAK2 immunoblotting reveals relative amounts of protein in each lane (lower panels). In B (right panels), total lysates are shown to demonstrate STAT3 is phosphorylated upon IL-10 treatment although did not co-precipitate with JAK2, and phosphorylation of the latter is not induced by IL-10. Similar results were obtained in three experiments.

To further demonstrate the involvement of JAK2 in contact-dependent STAT3 activation, we directly assessed JAK2 phosphorylation in cell extracts from the various treatments using SDS-PAGE and immunoblotting using anti-phosphotyrosine JAK2. A band corresponding to phosphorylated JAK2 appeared only when monocytes and tumor cells (MCF-7) were co-cultured together (Fig. 5A) but not when either conditioned media or IL-10, were used (Fig. 5B and C, respectively). As expected, this contact-dependent JAK2 phosphorylation was significantly reduced upon pretreatment of monocytes with JSI-124, paralleling that of phosphorylated STAT3 (Fig. 5A). Significantly, while no phosphorylated JAK2 was detected in either conditioned media or IL-10 treated monocytes, phosphorylated STAT3 was readily detected and was insensitive to JSI-124 treatment, (Fig. 5B and C and 6B). Of note, since only monocytes were pre-treated with the inhibitor JSI-124, the reduced level of phosphorylation of both JAK2 and STAT3 in the co-cultures strongly suggests that these events originate in the monocytes.

### phosphorylated-STAT3 co-immunoprecipitates with JAK2 upon MCF7:monocyte cell contact but not IL-10 treatment

Furthermore, we found that JAK2 and STAT3 are physically associated and that the latter was tyrosine-phosphorylated after contact-dependent stimulation using co-IP experiments. MCF-7 and monocytes were mixed together and were either lyzed immediately or after 2 hours incubation, followed by JAK2 immuno-precipitation. Phosphorylated-STAT3 was detected in JAK2 immunoprecipitates of MCF-7-monocyte co-cultures but not in control cells (Fig. 6A) or in IL-10 stimulated monocytes (Fig. 6B, left panel) although STAT3 phosphorylation is observed in the latter (Fig. 6B, right panel). Taken together, these data indicate that there are two separate mechanisms for the activation of STAT3 (soluble cytokines versus cell-cell contact) that differ in their usage of JAKs and their susceptibility to inhibition by JSI-124.

### DC exhibit a different phenotype upon cell:cell interaction as compared to IL-10 treatment

**Figure 7:**
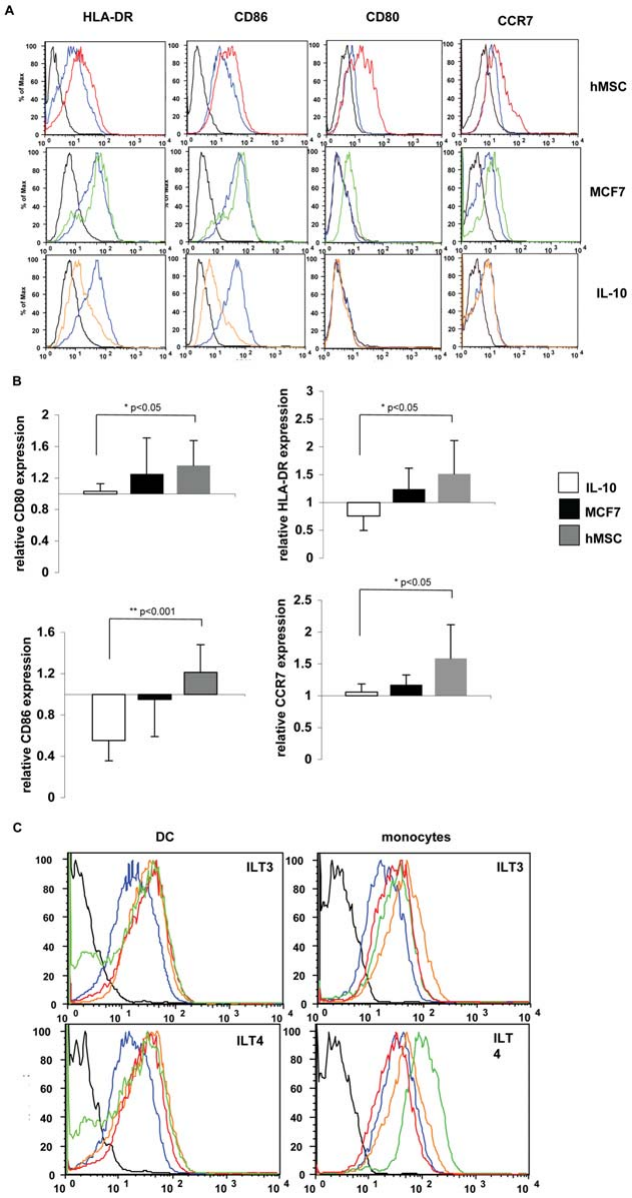
DCs exhibit altered matured phenotype upon cell:cell interaction but not after incubation with soluble factors. DCs were either incubated alone, co-cultured with hMSC (top panel) or MCF-7 (middle panel), or were treated with IL-10 (10 ng/ml; lower panel). After 24 hours, the cells were collected and the levels of HLA-DR, CD80, CD86 and CCR7 surface expression on DCs were determined by immunofluorescence and flow cytometry. Black line: isotype control; blue line DCs alone; Red line: DCs co-cultured with hMSC; Green line: DCs co-cultured with MCF-7; Orange line: DCs treated with IL-10 one representative experiment is shown (A). Mean fluorescent intensity (MFI) results as in A from 5 experiments were normalized and are presented as relative to the level of expression by DCs cultured alone (B). Either DCs or monocytes treated as above were further tested for surface expression of ILT3 and ILT4 by immunofluorescence and flow cytometry. Black line: isotype control; blue line: DCs or monocyes as indicated, alone; Red line: co-culture with hMSC; Green line: co-culture with MCF-7; Orange line: incubation with IL-10 (C).

Next we examined the effects of tumor secreted cytokines and cell-cell contact on APCs maturation. To test this, DCs were co-cultured with either MCF-7 cells or hMSC or alternatively treated with IL-10 for 24 h followed by examination of maturation markers by flow cytometry. Since MCF-7 cells induce STAT3 activation through both soluble factors and cell-cell contact, hMSC were included in this experiment given that their induction of STAT3 activity is solely dependent on cell-cell contact. As shown in figure 7, hMSC co-culturing had only a slight and statistically insignificant effect on the levels of HLA-DR and CD86 expression, while the levels of CD80 and CCR7 expression were significantly elevated (P = 0.007 and P = 0.003, respectively) compared to DCs single cultures. In contrast to cell-cell interaction, HLA-DR and CD86 levels were significantly reduced (P = 0.007 and P<0.001, respectively) by IL-10 treatment, whereas CD80 and CCR7 expression levels remained low (Fig. 7A). These results were quantified as shown in figure 7B and demonstrate a significant change in the pattern of maturation markers expression between IL-10 treated DCs (soluble) and co-cultures of DCs and hMSC (cell-cell contact), with the relative expression levels of CD86, HLA-DR, CD80 and CCR7 significantly differing between the two treatments. As expected, DC-MCF-7 co-cultures resulted in an intermediate phenotype between that observed in DC-hMSC co-cultures or in DCs treated with IL-10. This is consistent with the findings that MCF-7 cells can induce both soluble- and contact-dependent pathways in parallel. These findings represent a unique developmental stage for DCs upon cell-cell interaction that differs from the immature phenotype induced by IL-10.

### Dendritic cells and monocytes express the inhibitory receptors ILT3 and ILT4 upon interaction with MCF-7 cells, hMSC or IL-10

Immunoglobulin like transcript (ILT) are receptors structurally and functionally related to killer-cell inhibitory receptors (KIRs), containing long cytoplasmic tails containing immunoreceptor tyrosine based inhibitory motifs (ITIMs) that inhibit cell activation, and they have been shown to be involved in immune regulation. Specifically, ILT3 and ILT4 expression is limited to monocytes, macrophages and DCs. Expression of ILT3 and ILT4 on DC has been associated with a tolerogenic phenotype which was shown to be crucial to the tolerogenic capacity acquired by DCs [16], [17]. Interestingly, despite the marked differences in the overall phenotype between the contact- and soluble factor-dependent mechanisms the levels of expression of these two markers for tolerogenic APCs, ILT3 and ILT4 were upregulated in both DCs and monocytes co-cultured with hMSC or MCF-7 as well as when they were treated with IL-10 (Fig. 7C).

### hMSC:monocytes co-culture actively inhibit T-cell activation

Our previous results examining the immunomodulary effect of hMSC suggest that the cell-contact mechanism conferred immunoregulatory properties for APCs. Specifically, hMSC inhibited T-cell activation only upon addition of APCs (DC or monocytes) to the co-culture. The immunoregulatory properties acquired by these APCs upon contact included induction of IL-10 secretion together with reduction in TNF-α and IL-12 secretion by the APCs and inhibition of IFN-γ secretion and proliferation of T-cells [12]. In order to determine whether the contact-dependent mechanism converts the APCs into what is effectively a “deletional or inhibitory APC” with active immunoregulatory properties, as opposed to simply being activation incompetent, we examined the ability of these cell-contact conditioned APC to actively suppress T cell activation. In these experiments T cells were activated with anti-CD3 coated beads and soluble anti-CD28 that can stimulate T cells in the absence of any APCs and placed in the upper chamber of a transwell. Monocytes alone or in contact with hMSC were placed in the bottom chamber (to avoid contact between the monocytes and T cells that can contribute to T cell activation) and their effect on bead-stimulated T cell activation was determined by IFN-γ secretion. Here again hMSC were used and not cancer cells in-order to determine the exclusive contribution of the contact depended effect and to avoid the additional effect of soluble factor secreted by the cancer cells over the 72 h period. Inhibition of IFN-γ secretion was observed only when hMSC and monocyte were co-cultured in the lower chamber but not when each cell type was cultured alone (Supporting data Fig. S2). This data support our previous reports that upon cell-cell contact the APCs are converted into functionally regulatory cells via STAT3 activation and that this T cell inhibitory activity is partially mediated by a soluble factor secreted only upon cell-cell interaction [12], [13].

## Discussion

Cancer cells utilize different mechanisms to evade immune surveillance. One way is by influencing APCs within the tumor bed rendering them tolerogenic. Recent studies have shown that tumors secrete factors that inhibit DCs maturation through activation of STAT3 in DCs [7]. Here we show that in addition to releasing soluble factors, tumor cells, physically interact with APCs, consequently inducing the activation of STAT3. This induction results in APCs with a distinct phenotype, which are capable of inhibiting T-cell activation. Thus, tumor cells have at least two pathways by which they can alter APCs maturation and function. The first, described previously, is mediated by tumor-derived factors such as VEGF and IL-10, that promote the generation of immature DCs that lack the T cell stimulatory activity characteristic of mature DCs [7]. The second pathway, demonstrated in this study, is a novel cell contact induced and JAK2-dependent pathway that generates tolerogenic DCs with a distinct altered-mature phenotype that actively suppress T-cell activation. Hence, although the two pathways converge on activation of STAT3 they subsequently diverge in their downstream effect on APC phenotype.

In the current study, by simply washing the cells just prior to adding the APC we eliminated the contribution of tumor-secreted factors, thereby revealing the contact-dependent activation of STAT3. Second, we found that the cell contact-dependent pathway is sensitive to treatment with the JAK2 inhibitor JSI-124, distinguishing it from the soluble factor pathway. Third, phosphorylation of JAK2 and physical interactions between JAK2 and phosphorylated-STAT3 are contact dependent. These findings suggest that these are two distinct mechanisms for STAT3 activation that differ in their signaling pathways.

Besides cytokines and their receptors, STATs can be activated by other means, for example by growth factor receptors, such as EGF, PDGF, and CSF-1, which all possess an intrinsic tyrosine kinase activity [18], as well as by non-receptor tyrosine kinases such as Src and Abl, and through serpentine receptors such as those for angiotensin II, serotonin, and α-melatonin stimulating hormone [19], [4]. In addition, we have previously demonstrated, the induction of STAT3 signaling in APCs upon cell contact with hMSC, suggesting a new pathway for activating STAT3 in APCs. In a different setting, contact-dependent activation of STAT3 has been also demonstrated in normal and cancer breast cells. A study by Vultur et. al. showed a dramatic increase in STAT3 phosphorylation and consequently STAT3 activity with cell density in normal cells. This confluence-dependent activation was eliminated if cell-cell interactions were interrupted through calcium chelation [20]. The contact dependent activation of STAT3 described by Vulture et.al may be due to a mechanism similar to the one we describe here for a heterologous interaction between APCs and their cellular environment, suggesting that a contact dependent activation of STAT3 may be a more general mechanism. Taken together, one can speculate the existence of yet unknown surface molecules participating in intercellular contact that are dependent on JAK2 for their activity. The finding of such surface molecules requires further investigation.

DCs play a key role in the initiation and regulation of the immune response, and their ability to initiate immune reaction or induce tolerance is strictly dependent on their maturation state and phenotype. While immature DCs can induce T-cell anergy, mature DCs activate T cells and induce immunity. Nonetheless, it has also been reported that DCs may exist in a semi-mature state in which they are capable of actively inhibiting T cell activity, generating regulatory T-cell and promoting tolerance [2]. In this report, we found that contact-dependent activation of STAT3 induced the generation of functionally regulatory DCs with a seemingly semi-mature or aberrantly-mature phenotype, whereas IL-10 treatment resulted in DCs that express low levels of costimulatory molecules and HLA-DR, consistent with an immature phenotype. This data is in agreement with previous studies that described DCs with regulatory function that varied in their maturation phenotypes [21], [22], [23]. Among these are two reports describing the generation of regulatory DCs originating from mature cells rather than from immature cells [7], [24]. Therefore, it seems that the hallmark of regulatory DCs is their ability to functionally inhibit lymphocyte activation rather than their specific phenotype and maturation stage. In agreement with this notion, we further demonstrated that despite the different phenotypic outcomes, both IL-10 and cell-cell contact induced the expression of the inhibitory receptors Immunoglobulin like transcript (ILT) 3 and 4, which are characteristic of regulatory DCs, and play a role in their T cell inhibitory activity. Hence, ILT3 and ILT4 expression may account for the inhibitory activity of these cells regardless of their overall phenotype.

In response to an inflammatory stimuli, DCs up-regulate the expression of the chemokine receptor, CCR7 and migrate from peripheral tissues to secondary lymphoid organs [25]. While this event is usually linked to the process of DC's maturation, a previous study has demonstrated that after exposure to apoptotic cells DCs up-regulated CCR7 expression despite the absence of maturation properties [26]. Hence, the authors further speculated that CCR7-expressing immature DCs that ingested apoptotic material may migrate to peripheral lymph nodes, in order to maintain tolerance [26]. In our study we show that contact-dependent induced regulatory DCs up-regulate the expression of CCR7 thereby enabling these DCs to migrate to lymphoid tissues where they may induce tolerance. Notably, CCR7 is induced only by the contact-dependent mechanism and not by soluble factors, further emphasizing the potential importance of the contact-dependent mechanism in tolerance induction, such as in the case of tumors.

In the context of cancer, modulating APCs in a contact-dependent manner may be of special significance in the pre-malignant stage. Physiologically, tumors arise sporadically and between the initiating oncogenic event and tumor progression there might be a long pre-malignant stage. However, data concerning the interaction between tumor cells and the immune system was obtained from models in which the host was exposed to a large number of cells at a single time point. A recent study by Willimsky et.al. [27], has addressed the question of immunogenicity of pre-malignant lesions, by using a transgenic mouse model for sporadic cancer. The authors of this study concluded that tolerance occurs already at the pre-malignant stage, before tumor progression. Specifically, they showed the accumulation of immature myeloid cells at the pre-malignant stage, and suggest they play a role in creating T-cell unresponsiveness. Given our results, one may speculate that at the pre-malignant stage physical interactions between resident APCs and tumor cells that activate STAT3 play a significant role in tumor escape, long before tumors are visible and large enough to secrete significant amounts of APCs-modulating factors.

## Materials and Methods

### Cells

Cells were purified from the venous blood of healthy donors. Either, CD4^+^ T cells or CD14^+^ cells were isolated by negative selection using the RosetteSep™ enrichment cocktail (StemCell Technologies, Vancouver, Canada). hMSC were obtained from discarded bone tissues from patients undergoing total hip replacement surgeries, under approval of Hadassah Medical Center Helsinki Ethics Committee following an informed consent. The hMSC were separated from other bone-marrow residing cells by plastic adherence, and were then grown under tissue culture conditions, as previously described [13]. The cells were maintained in a low-glucose DMEM medium supplemented with 10% heat-inactivated fetal calf serum, 2 mM glutamine, and penicillin/streptomycin (Biological Industries, Beit-Haemek, Israel). For DC generation, CD14^+^ cells were plated in RPMI 1640 (Biological Industries) containing 1% autologous plasma, 0.1 µg/ml IL-4 and 0.1 µg/ml GM-CSF (PeproTech, Rocky Hill, NJ). Every 2 days 0.3 ml were removed and 0.5 ml media containing plasma and cytokines were added. By day 7, >90% of the cells were CD14^-^ and CD11c^+^ immature DCs. For JAK blockade freshly isolated CD14^+^ cells or DC were pre-incubated with JAK2 inhibitors, JSI-124 (cucurbitacin I; Calbiochem,). Human breast adenocarcinoma (MCF7) and human prostate adenocarcinoma (PC3), cell lines were obtained from the American Type Culture Collection (ATCC). MCF7 cells were cultured in DMEM with 10% heat-inactivated fetal calf serum, 2 mM glutamine, and penicillin/streptomycin (Biological Industries, Beit-Haemek, Israel) at 37°C and 5% CO_2_. PC3 cells were cultured in Roswell Park Memorial Institute (RPMI) 1640 medium (Biological Industries, Beit-Haemek, Israel) supplemented with 10% heat-inactivated fetal calf serum (Biological Industries), 2 mM glutamine, and penicillin/streptomycin, at 37°C and 5% CO_2_.

### Cell lysis and immunoblotting

Either tumor cells and APC (either monocytes or DCs) alone, or the mixture of the two were incubated at 37°C. After 2 hours, whole cell extracts were prepared using lysis buffer (0.5% Nonidet P-40, 50 mM Tris-HCl (pH 8.0), 100 mM NaCl, 1 mM PMSF, 1 mM sodium orthovanadate, 10 µg/ml leupeptin, and 10 µg/ml aprotinin) for 20–30 min on ice. Lysates were separated by electrophoresis on 10% SDS-PAGE gels and then transferred to PVDF membranes (Bio-Rad, Hercules, CA). The blots were probed with anti-phosphorylated STAT3 mAb (Tyr705; Cell Signaling, Danvers, MA) or anti-phosphorylated Jak2 mAb (Tyr1007/1008; Cell Signaling, Danvers, MA) processed with ECL plus Western Blotting detection system (Amersham- Pharmacia Biotech), and exposed to Chemiluminescence BioMax Light Film (Kodak-Industries, Cedex, France). Following stripping the membranes were re-probed with anti-STAT3 mAb (Santa-Cruz Biotechnology, Santa-Cruz, CA) or anti-JAK2 Rabbit mAb (Cell Signaling, Danvers, MA).

### STAT3 transcription factor activity assay

Tumor cells and APC (either monocytes or DC) were incubated either alone or co-cultured together for 2 hours and then nuclear extracts were prepared using Nuclear Extraction kit (Chemicon International, Temecula, CA). The lysates were then used to determine the specific binding of STAT3 to their respective targets using a STAT3 transcription factor assay kit (Chemicon International,) and the results were confirmed with the Human Active STAT3 DuoSet IC kit (R&D systems, Minneapolis, MN). Specificity was determined using non-specific oligomer as well by competing the binding with non-biotinylated oligomer supplied in the kit.

### Cytokine production

Cultures containing 5×10^4^ CD4^+^ T cells and 5×10^4^ monocytes in the presence of the superantigen SEB (1 ng/ml; Sigma, St. Louis, MO), and in the absence or presence of tumor cells (5×10^4^), were plated in individual wells of flat-bottom 96-well plates (Corning, Corning, NY). Cells were stimulated for 72 hours and conditioned media were collected. IFN-γ levels in the conditioned media were assayed by ELISA (R&D Systems). IL-10 secretion was determined in the conditioned media of either monocytes, tumor alone or co-culture of the two using commercial ELISA (R&D Systems).

### Intracellular staining and Flow cytometry

Monocytes and tumor cells were cultured alone or mixed together for 24 hours, with the addition of 2 µM monensin for the last 5 hours of stimulation. Then the cells were harvested, washed twice in PBS, fixed in 4% paraformaldehyde for 30 minutes, washed twice in PBS 1% FCS, resuspended in 0.1% saponin, 1% FCS/PBS, and stained using PE-conjugated anti-cytokine IL-10 (Serotec, Oxford, UK) or anti-phosphorylated STAT3 antibodies (Santa-Cruz Biotechnology), for 30 minutes. Cells were washed twice in PBS, and the immunostained cells (1×10^4^ cells/sample) were analyzed on a FACS Calibure flow cytometer (Becton Dickinson, San Jose, CA) using the Cell Quest software.

### Immunoprecipitation

For immunoprecipitations, whole cell lysates (prepared as described above) were precleared for 1 h with protein A-sepharose beads (Sigma-Aldrich St. Louis, MO, USA), and then incubated with the anti-JAK2 Rabbit mAb (Cell Signaling) for at least 1 h and then protein A-Sepharose beads were added and incubated o.n with gentle rotation. Beads were then washed three times in 500 µl of lysis buffer. Samples were resuspended in reducing SDS sample buffer, heated at 95°C for 5 min, and separated by SDS-PAGE, and proteins were transferred to PVDF membrane (Bio-Rad, Hercules, CA) for analysis by Western blotting.

### APC phenotype

DCs were cultured for 24 hours in the absence or presence of hMSCs, tumor cells or IL-10. CD86 (PharMingen, BD Biosciences, Mississauga, ON. Canada), human leukocyte antigen-DR (HLA-DR) (Serotec), CD80 (Biolegend San Diego, CA), CCR7 (BD Biosciences, Mississauga, ON. Canada), ILT3 (R&D Systems) and ILT4 (R&D Systems) expression was measured by direct immunofluorescence using conjugated antibodies, and the immunostained cells were analyzed on a FACSCaliber flow cytometer (Becton Dickinson, San Jose, CA) using CELLQuest software (Becton Dickinson). The data were calculated as the mean Fluorescence intensity.

### APC function

CD4^+^ T-cells were stimulated with anti-CD3 mAb (OKT3; eBioscience San Diego, CA, USA) immobilized on protein A-sepharose beads (Sigma-Aldrich) in combination with soluble anti-CD28 mAb (0.5 µg/ml;R&D Systems) and placed in the upper chamber of a transwell. Either monocytes, hMSC alone or in combination were placed in the lower chamber. After 72 hr. medium was collected and IFN-γ levels in the conditioned media were assayed by ELISA (R&D Systems).

## Supporting Information

Figure S1 Cancer cells inhibit TNF-α and IFN-γ secretion while inducing IL-10 production. A: Monocytes were used to activate autologuos CD4+ T cells (each 5×104 cells per well) with SEB (1 ng/ml) in the presence or absence of either MCF-7 (upper panels) or PC3 (lower panels; 5×104 cells), in triplicate wells of a 96 well dish. After 72 hours, conditioned media were collected and the level of IFN-γ and TNF-α were determined using ELISA. The data represent the mean values of triplicate samples and standard deviations. Data represents one of three experiments. B: Upper panels: monocytes and PC3 were cultured alone or were co-cultured together in triplicate wells of a 96 well dish. After 72 hours, conditioned media was collected and the level of IL-10 was determined using ELISA. The data represent the mean values of triplicate samples and standard deviations. Lower panels: Monocytes and either MCF-7 or PC3 were cultured alone or were co-cultured together. After 24 hours, the cells were harvested and IL-10 was detected by intracellular immunostaining and flow cytometric analysis. Monocytes that were gated based on their forward- and side-scatter characteristics are shown. Dashed line: isotype control; Filled grey area: monocytes alone; Black line: tumor cells:monocytes co-culture; Comparable results were obtained in three separate experiments.
(0.11 MB TIF)

Figure S2 hMSC conditioned monocytes actively inhibit T-cell activation. T cells were activated with anti-CD3 coated beads and soluble anti-CD28 and were placed in the upper chamber of a transwell. Monocytes, hMSC or their co-culture were placed in the bottom chamber. After 72 hours, conditioned media were collected and the level of IFN-γ was determined using ELISA. The data represent the mean values of triplicate samples and standard deviations. Data represents one of three experiments.
(0.07 MB TIF)
