# Supplementary figures and images for: The Induction of APC with a Distinct Tolerogenic Phenotype via Contact-Dependent STAT3 Activation
Source: PLoS One. 2009 Aug 31;4(8):e6846. doi: 10.1371/journal.pone.0006846 (PMC2731174; doi:10.1371/journal.pone.0006846)

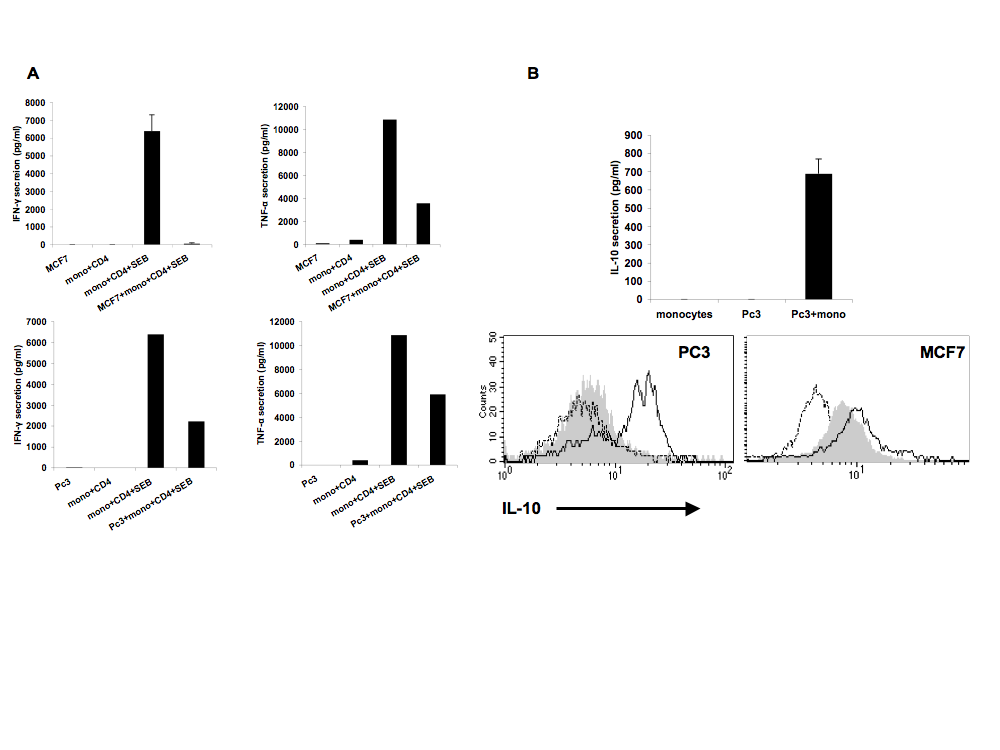

Supplement: Figure S1 — Cancer cells inhibit TNF-α and IFN-γ secretion while inducing IL-10 production. A: Monocytes were used to activate autologuos CD4+ T cells (each 5×10^4^ cells per well) with SEB (1 ng/ml) in the presence or absence of either MCF-7 (upper panels) or PC3 (lower panels; 5×10^4^ cells), in triplicate wells of a 96 well dish. After 72 hours, conditioned media were collected and the level of IFN-γ and TNF-α were determined using ELISA. The data represent the mean values of triplicate samples and standard deviations. Data represents one of three experiments. B: Upper panels: monocytes and PC3 were cultured alone or were co-cultured together in triplicate wells of a 96 well dish. After 72 hours, conditioned media was collected and the level of IL-10 was determined using ELISA. The data represent the mean values of triplicate samples and standard deviations. Lower panels: Monocytes and either MCF-7 or PC3 were cultured alone or were co-cultured together. After 24 hours, the cells were harvested and IL-10 was detected by intracellular immunostaining and flow cytometric analysis. Monocytes that were gated based on their forward- and side-scatter characteristics are shown. Dashed line: isotype control; Filled grey area: monocytes alone; Black line: tumor cells:monocytes co-culture; Comparable results were obtained in three separate experiments. (0.11 MB TIF) [file pone.0006846.s001.tif]

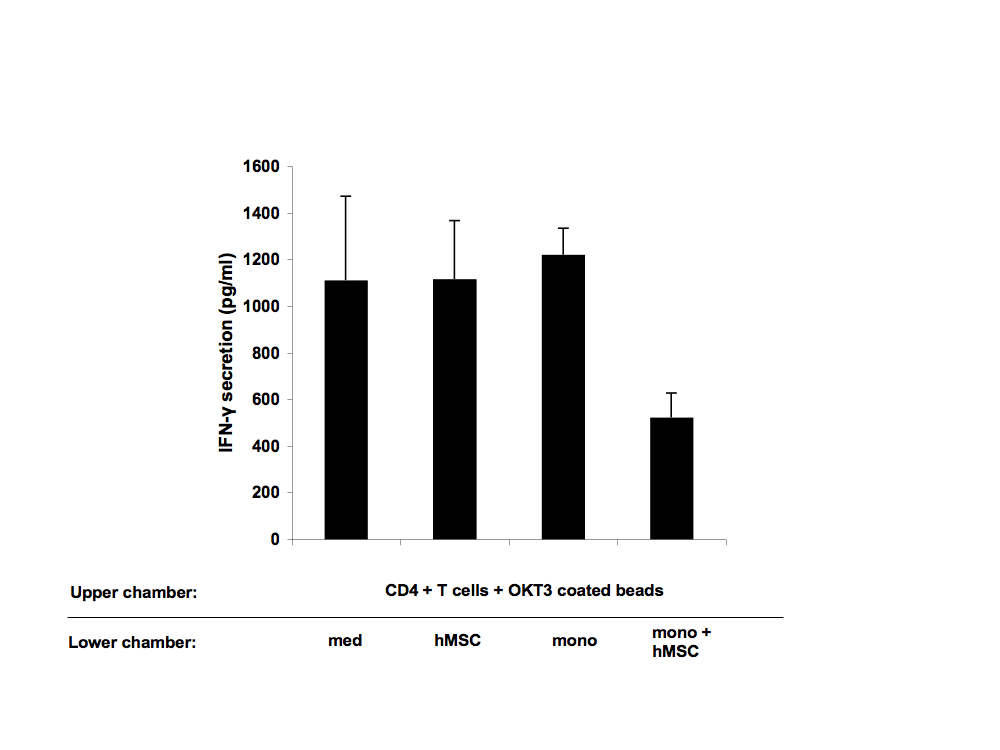

Supplement: Figure S2 — hMSC conditioned monocytes actively inhibit T-cell activation. T cells were activated with anti-CD3 coated beads and soluble anti-CD28 and were placed in the upper chamber of a transwell. Monocytes, hMSC or their co-culture were placed in the bottom chamber. After 72 hours, conditioned media were collected and the level of IFN-γ was determined using ELISA. The data represent the mean values of triplicate samples and standard deviations. Data represents one of three experiments. (0.07 MB TIF) [file pone.0006846.s002.tif]
